# Comparative Growth and Development of Spiders Reared on Live and Dead Prey

**DOI:** 10.1371/journal.pone.0083663

**Published:** 2013-12-27

**Authors:** Yu Peng, Fan Zhang, Shaolan Gui, Huping Qiao, Grant C. Hose

**Affiliations:** 1 College of Life Sciences, Hubei University, Wuhan, Hubei, P.R. China; 2 Department of Biological Sciences, Macquarie University, Sydney, New South Wales, Australia; United States Department of Agriculture, Beltsville Agricultural Research Center, United States of America

## Abstract

Scavenging (feeding on dead prey) has been demonstrated across a number of spider families, yet the implications of feeding on dead prey for the growth and development of individuals and population is unknown. In this study we compare the growth, development, and predatory activity of two species of spiders that were fed on live and dead prey. *Pardosa astrigera* (Lycosidae) and *Hylyphantes graminicola* (Lyniphiidae) were fed live or dead fruit flies, *Drosophila melanogaster.* The survival of *P. astrigera* and *H. graminicola* was not affected by prey type. The duration of late instars of *P. astrigera* fed dead prey were longer and mature spiders had less protein content than those fed live prey, whereas there were no differences in the rate of *H. graminicola* development, but the mass of mature spiders fed dead prey was greater than those fed live prey. Predation rates by *P. astrigera* did not differ between the two prey types, but *H. graminicola* had a higher rate of predation on dead than alive prey, presumably because the dead flies were easier to catch and handle. Overall, the growth, development and reproduction of *H. graminicola* reared with dead flies was better than those reared on live flies, yet for the larger *P. astrigera,* dead prey may suit smaller instars but mature spiders may be best maintained with live prey. We have clearly demonstrated that dead prey may be suitable for rearing spiders, although the success of the spiders fed such prey appears size- and species specific.

## Introduction

As obligate predators, spiders play important roles in terrestrial food webs but consequently are susceptible to environmental changes either directly or via changes in prey populations. As a result, spiders may be particularly sensitive to environmental perturbations and are useful bioindicators of environmental conditions [1]. Spiders also have a long history of beneficial uses for humans, including medicinal use [2], and more recently as sources of antimicrobial compounds [3], [4]. For these benefits, the laboratory rearing of live spiders is necessary to achieve a critical population size and biomass.

The long held paradigm that the diet of spiders is restricted to live prey [5] has been challenged by recent research, which has demonstrated ‘scavenging’ feeding behavior across a number of spider families [6], [7]. While spiders are generally known for feeding on other active arthropods, some eat a variety of non-moving prey such as the eggs of insects or other spiders [8]–[11], and non-prey foods, such as honeydew, pollen, nectar [12], [13], and plants [14]–[16].

Despite this emerging breadth of non-living components in spider diets, many spiders do require live prey [5], and, although a large number of species have been examined in this regard [6], [7], the dietary preferences of most species is unknown. Furthermore, research to date has examined the short-term live/dead feeding preferences of individuals, yet there is a need to determine whether non-living prey can be used as a full substitute for live prey in spider diets without effects on spider growth and population viability.

Based on the live-prey feeding paradigm, the culture of spider populations *in vivo* has to date, required the concurrent raising and feeding of live prey items. With difficulties in maintaining live prey populations, we examined the potential for using dead prey as a substitute, such that laboratory populations can be reared in a less labor intensive manor. Accordingly, in the present paper, we consider three questions: 1) Can spiders be reared on dead prey? 2) How does the dead prey effect the growth, development, reproduction of the spiders in the laboratory? 3) Does predacious ability of spiders change when fed live or dead prey? We address these questions and compare the response of two different spider species.

## Materials and Methods

### Spiders and Rearing

*Pardosa astrigera* (Araneae: Lycosidae) and *Hylyphantes graminicola* (Araneae: Linyphiidae) were chosen for the study. The two species of spiders are common and abundant in the agricultural fields of China [17]. *Pardosa astrigera* is a medium size (body length 5.5–10.0 mm) wandering spider. *Hylyphantes graminicola* is a smaller (length 2.5–3.5 mm), web-weaving species. *Pardosa astrigera* with egg sac in spinnerets was collected from fields in the Huazhong Agricultural University, Wuhan (114°31′N, 30°52′E), China, from April to May 2009. Adult female *H. graminicola* were collected from the corn fields in the Huazhong Agricultural University in Wuhan, China. No specific permissions were required for land access or spider collections. This study did not involve or otherwise interfere with any endangered or protected species.

Spiders were kept individually in cylindrical glass tubes (diameter × height = 2×12 cm) and acclimated to laboratory conditions (relative humidity: 80–85%; temperature: 25±1°C; light regime: 12∶12 h; lights on at 08∶00) for five days during which they were not fed. After acclimation, the spiders were fed once every two days on living adult fruit flies (*Drosophila melanogaster*). Water and sugar water were provided continuously via moistened cotton rolls. The 2^nd^ instar spiderlings of *P. astrigera* were collected after dispersing from the abdomens of maternal spiders. The 2^nd^ instar spiderlings of *H. graminicola* were collected approximately 5 days after the 1^st^ instar spiderlings had molted.

### Prey Culturing

*Drosophila melanogaster* was used as prey in these experiments because it is a common food source for laboratory-reared animals, can be cultivated easily in the laboratory, and is a likely prey for spiders in the natural environment. Flies were reared at 22–24°C with a 12-h light-dark regime, and the standard cornmeal diet. Flies were killed by placing them in glass tubes in a −20°C freezer for one hour. Dead flies were removed from the freezer and brought to room temperature before being made available to the spiders.

### The Effect of Prey on the Survival, Growth and Development of Spiders

Second instar spiderlings of *P. astrigera* and *H. graminicola* were used for this experiment. Two hundred spiderlings of each species were collected and randomly assigned to either of two groups. The first group was fed with living flies, the second group were fed with dead flies. Flies were provided in excess, and the number of flies increased with the increasing instars of the spiders. The death and molt of spiderlings were recorded daily. When spiders matured, the fresh mass of the whole spider was recorded.

### Predation of Adult Spiders on Live and Dead Flies

The rate of predation of adult female *P. astrigera* and *H. graminicola* was investigated after the spiders were starved for 48 h. For *P. astrigera,* adult females were chosen randomly and kept individually in glass tubes (diameter × length = 3.5×10 cm). Spiders were fed with either 10, 15, 20, 25 or 30 living or dead flies that were placed into the tubes. The numbers of flies preyed upon by the spiders was recorded after 24 h. Each density of flies had three replicates. The predatory response was calculated according to Holling [18].

For *H. graminicola,* spiders were randomly allocated to the similar glass tubes and fed either 10 live or dead flies. The number of flies that were eaten by the spiders within 12 h was recorded. For dead flies, the spiders were watched continually over the 12 h period and the specific flies that were eaten was noted, and return visits to eat the same fly were not counted.

### Analysis of Protein Content of Adult *P. astrigera*

Six adult female *P. astrigera* were randomly sampled from the dead and live fly treatments. Each group had three replicates. Spiders were homogenized with phosphate buffer (0.1 M, pH 7.5) and centrifuged (15000 g for 10 min), the supernatants used for the estimation of protein content. Protein concentrations in the spiders were estimated using the colourimetric method of Bradford [19]. Coomassie Brilliant Blue G-250 dye was dissolved in 95% ethanol and 85% H_3_PO_4_. Five millilitres of this reagent solution was added to 0.1 mL of protein sample. After 2 minutes, the absorbance of the solution was measured at 595 nm by using a Beckman DU-40 spectrophotometer. Analytical blanks were made by replacing the protein solution with 0.1 mL of the reagent solution.

### Analysis of Fecundity of *H. graminicola*

The fecundity of female *H. graminicola* which were reared to maturity by feeding only live or dead flies was compared. Female *H. graminicola* were randomly paired with a mature male and left together for 48 h to mate. Each treatment (live and dead prey) had 30 replicates. The time of subsequent egg laying, the number of egg sacs laid, the total number of eggs per sac and hatching rate of the eggs were observed and recorded for each pair.

### Statistical Analyses

Differences of means between treatments (live and dead prey) were compared by using t-tests. All calculations were carried out in SPSS version 11.5 software package (SPSS Inc). The significance level (α) for all tests was 0.05.

## Results

### The Effect of Prey on Spider Survival

**Figure 1:**
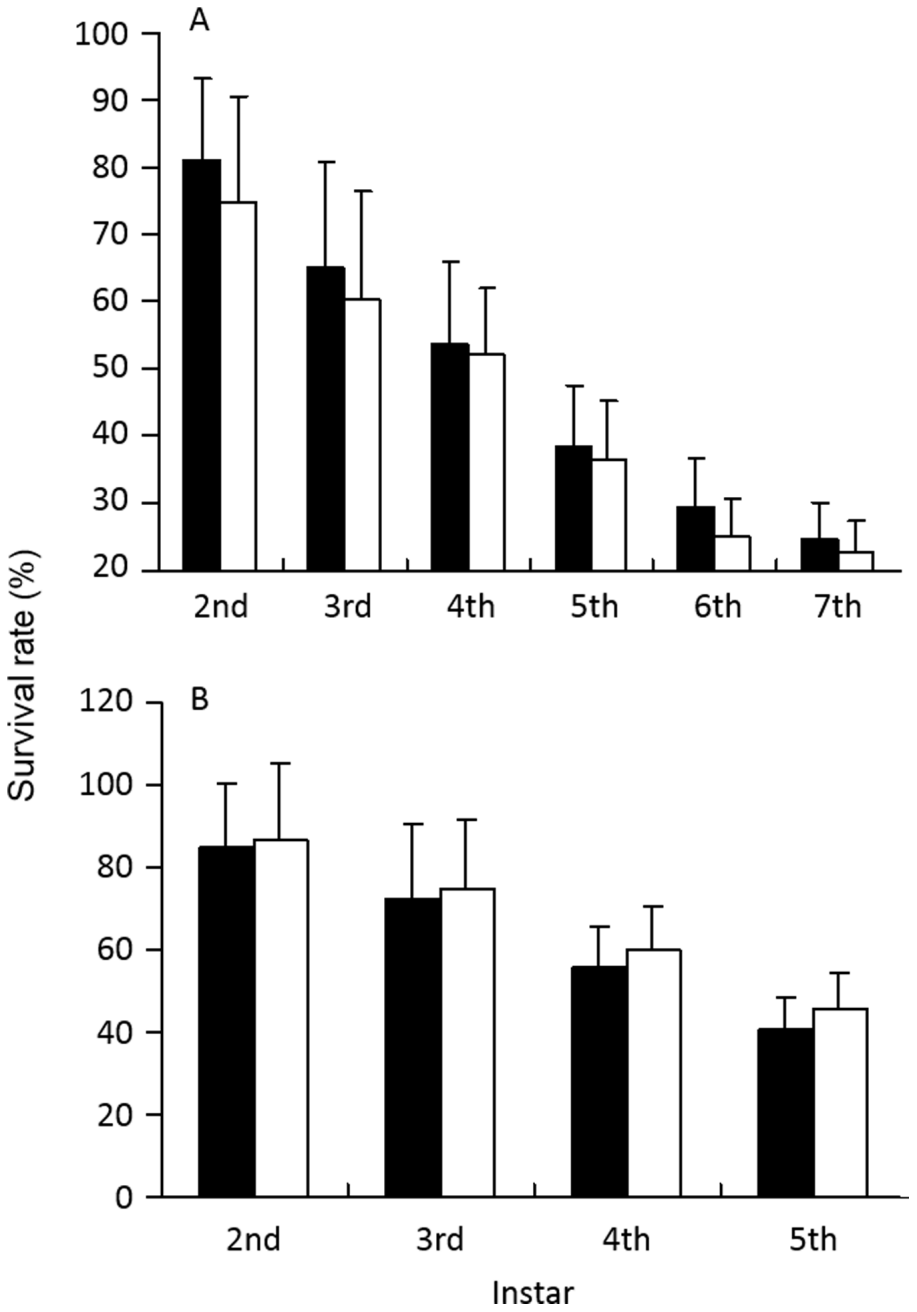
Survival rate of a) *Pardosa astrigera* and b) *Hylyphantes graminicola* reared with live (shaded bars) and dead (white bars) prey.

The survival of both species declined with increasing instar (Fig. 1a, b), however, there was no significant difference in the survival of each species and each instar with food source (p>0.05).

### The Effect of Prey on Spider Development

The time to maturity of *P. astrigera* reared with dead flies was significantly longer than that for spiders fed live flies (Table 1). The durations of early instars was similar between the treatments but the duration of the 5th and 6th instars was significantly longer for spiders fed dead flies than for those fed live flies (Table 1). There was no significant difference in the durations for each instar and total developmental periods for *H. graminicola* fed live or dead flies (p>0.05).

**Table 1:** Average developmental period (days± SE) of *Pardosa astrigera* and *Hylyphantes graminicola* fed with live and dead flies as prey.

Spiders	Instar	Live prey	Dead prey	t-value	p
*Pardosa astrigera*	2nd	12.8±0.5	13.4±0.5	−2.125	0.353
	3rd	11.6±0.7	12.2±0.6	−1.673	0.416
	4th	13.5±0.5	14.1±0.7	−0.307	0.104
	5th	14.5±0.6	16.8±0.3	−2.167	0.043
	6th	15.2±0.5	17.9±0.5*	−3.384	0.020
	Total	62.7±0.4	76.4±0.9**	−6.318	<0.001
*Hylyphantes graminicola*	2nd	11.3±0.6	10.7±0.6	0.734	0.473
	3rd	12.4±0.5	11.5±0.5	1.221	0.238
	4th	12.7±0.7	12.2±0.6	0.576	0.571
	5th	13.7±0.7	12.9±0.7	0.823	0.421
	Total	48.7±0.9	46.8±0.7	1.676	0.111

### Predation by Adult Spiders

There was no significant difference in the predation rate of *P. astrigera* fed live or dead flies across the five prey densities. Predation increased with fly density in both live and dead prey treatments (Table 2) corresponding with Holling’s type II functional response (Table 3). In contrast to *P. astrigera*, the predation rate of *H. graminicola* on dead flies was significantly greater than that on live flies (p = 0.002).

**Table 2:** The average number (±SE) of live and dead flies preyed upon by *Pardosa astrigera* at increasing prey densities.

Prey density(Flies per tube)	Amount of flies preyed by *P. astrigera*	
	Live prey	Dead prey
10	9.4±0.4	9.0±0.4
15	12.0±0.3	11.2±0.4
20	13.2±0.4	12.6±0.2
25	13.8±0.4	13.2±0.4
30	14.8±0.4	13.6±0.2

**Table 3:** Functional response parameters of *Pardosa astrigera* reared to maturity with live and dead prey.

Foods	Functional response equation	N_amax_	T_h_ (d)	a	R
Live prey	1/N_a_ = 0.6151/N+0.0454	22.0	0.0454	1.6258	0.9914
Dead prey	1/N_a_ = 0.5935/N+0.0514	19.5	0.0514	1.6849	0.9911

### Effect of Prey on the Protein Content of *P. astrigera*

*Pardosa astrigera* that were reared to maturity with dead flies (7.5±0.2 mg/mL) contained significantly less (p = 0.002) protein than spiders reared on living flies (9.7±0.3 mg/mL).

### Effects of Prey on Body Mass of *H. graminicola*

The body mass of male and female spiders fed with dead flies was not significantly different from those of spiders of the same gender fed live flies (p = 0.143, 0.104, respectively).

### Effect of Prey on Fecundity of *H. graminicola*

Spiders reared with dead flies had significantly shorter pre-oviposition intervals (defined as the average time from copulation to the first oviposition), and produced a greater number of eggs than those reared on living flies (Table 4). There was no significant difference in the rate of hatching or the number of egg sacs produced between *H. gramminicola* fed live or dead flies (Table 4).

**Table 4:** The average fecundity (±SE) of *Hylyphantes graminicola* reared with dead flies.

Items	Live prey	Dead prey	t-value	p
Pre-oviposition intervals	10.1±0.4**	7.6±0.3	4.913	<0.001
Number of egg sacs	2.8±0.3	3.4±0.3	−1.640	0.118
Total number of egg	31.3±2.8	40.1±2.4*	−2.676	0.015
Hatching rate	79.4±1.0	80.3±1.2	−0.578	0.571

## Discussion

This study demonstrates that feeding on solely either live or dead prey can influence the growth and development of spiders but that the response is species specific. There was little effect of prey type (alive or dead) on the growth and development of *H. graminicola.* Although more dead than live flies were preyed upon, it is unclear whether this translates to an increase in consumption. Evidence suggests *H. graminicola* populations may be successfully reared on dead prey. For *P. astrigera*, there is evidence to suggest that rearing this species on dead flies may be suboptimal, particularly for later instars. Spiders reared on dead flies took longer to develop and had significantly lower body-protein concentrations than those fed live flies, suggesting a decrease in fitness of those animals.

The greater predation of *H. graminicola* on dead rather than living flies is likely a function of the relatively small size of this species compared to the prey. The handling of living prey by this small spider is undoubtedly difficult, and hence the preference for the dead prey that requires lesser effort. In contrast, for the larger *P. astrigera*, for which the handling effort is less of an issue, there was no difference in the feeding rate upon live and dead flies at the same prey density.

It is usual that non web-building spiders kill more prey than they consume [20], which makes it difficult to determine the relative consumption of the live and dead prey. However, at least for *Pardosa* spp., it is likely that partial consumption of prey may not occur until the spider is close to satiation [21]. In this study, we found no difference in the predation of *P. astrigera* on the live or dead prey, yet those spiders fed dead prey had a lower protein content and took longer to develop than those fed live prey. This indeed suggests greater nutritional value of the live compared to the dead prey. Interestingly, *Pardosa milvina* spiders offered low and high quality flies had a higher rate of predation on the poorer quality flies [22], but such relationships to food quality were not evident in our study.

*Hylyphantes graminicola* fed dead flies did not differ in developmental time from those fed live flies. The time to maturation recorded in this study (∼47 days) was somewhat longer than that reported previously [23], [24]. In contrast, the development of *P. astrigera* was slowed by rearing on dead spiders. The development of *P. astrigera* was also slower than reported for this species previously (49 days, [1]), and the increase in developmental time is similar to that caused by chemical exposure in this and other *Pardosa* spp. [1], [25]. However, although the overall development of *P. astrigera* was slowed, it was only the duration of the later instars that differed significantly with prey type (Table 1). This suggests that for smaller, early instar spiders, there is no differences in prey type on development, and highlights the age specific food requirements seen in many spiders [26]. Just as for *H. graminicola*, we hypothesized that this is due to the ability of the smaller spiders to more easily handle the dead flies. We expect that the similar growth of the spiders fed live and dead prey reflects a trade off in which spiders are able to feed on more, but lower quality dead flies compared to fewer, higher quality living flies.

Interestingly, despite there being greater predation of dead flies by *H. graminicola*, this did not translate to greater body mass, which suggests that despite attacking more dead than alive individuals, the overall consumption of prey did not change. This may reflect either lesser consumption of each prey item, or predation to satiation on a small number of individuals and subsequently, non-consumptive attacks on other dead flies, which is consistent with the expectation of spiders killing more prey than they consume [20].

The type II functional response for *P. astrigera* is consistent with that determined previously for lycosid spiders in general and *Pardosa* species in particular [22]. In a type II functional response, spider consumption of prey increases with prey density until a plateau is reached [28]. Interestingly, Schmidt et al. [22] determined similar type II functional responses for *P. milvina* fed *Drosophila* of different nutritional qualities. In this study also, we determined type II functions for *Pardosa* sp. fed live and dead prey, which, based on the resulting spider mass may well be of different quality. Surprisingly also, the feeding rate of *P. astrigera* was not different for the live and dead prey. Presumably, for *Pardosa* sp. that are ambush predators, the time required to capture dead prey, and the subsequent handling time would be less than that for live prey. Nevertheless, there was no difference in the predation of the spider on either prey.

Fruit flies (*Drosophila* sp.) are used as food for many kinds of spiders, such as thomisids, clubionids, oxyopids, lycosids, araneids, theridiids and linyphiids [17]. They are readily cultured and grown under laboratory conditions but are more difficult to transport and maintain outside the laboratory. In such situations, the use of dead flies that can be pre-prepared represents a viable alternative. Just as bees are maintained and transported around agricultural regions to promote pollination, an ability to maintain spider populations raises the possibilities that spiders may, in future, be reared on site and released when needed to control pest outbreaks. The importance of *H. graminicola* to natural pest control is well known [16] and our research here opens the possibilities for the establishment of spider cultures without the concurrent need for a live food source.

Overall, laboratory populations of *H. graminicola* are likely to be improved in terms of individual spider condition and population growth by feeding with dead *Drosophila*, because it is easier for the small spider to handle the dead prey. In contrast, there appears little benefit to the larger *P. astrigera.* Indeed, there is a decrease in spider condition and populations take longer overall to increase. Our study provides evidence of the need for species- and size-specific selection of prey for spider rearing. This study also provides further evidence of scavenging behavior in spiders and suggests that such feeding behavior can, for some species, be the sole means of food gathering without effects on development or a reduction in individual or population growth.
